# *N* ^6^-methyladenosine RNA methylation: From regulatory mechanisms to potential clinical applications

**DOI:** 10.3389/fcell.2022.1055808

**Published:** 2022-11-03

**Authors:** Peipei Li, Yuntao Wang, Yiwen Sun, Sanjie Jiang, Jingjing Li

**Affiliations:** ^1^ Department of Oncology, Weifang Medical University, Weifang, China; ^2^ BGI Genomics, BGI-Shenzhen, Shenzhen, China

**Keywords:** RNA methylation, *N* ^6^-methyladenosine, regulatory mechanisms, cancer, tumor therapy

## Abstract

Epitranscriptomics has emerged as another level of epigenetic regulation similar to DNA and histone modifications. *N* ^6^-methyladenosine (m^6^A) is one of the most prevalent and abundant posttranscriptional modifications, widely distributed in many biological species. The level of *N* ^6^-methyladenosine RNA methylation is dynamically and reversibly regulated by distinct effectors including methyltransferases, demethylases, histone modification and metabolites. In addition, *N* ^6^-methyladenosine RNA methylation is involved in multiple RNA metabolism pathways, such as splicing, localization, translation efficiency, stability and degradation, ultimately affecting various pathological processes, especially the oncogenic and tumor-suppressing activities. Recent studies also reveal that *N* ^6^-methyladenosine modification exerts the function in immune cells and tumor immunity. In this review, we mainly focus on the regulatory mechanisms of *N* ^6^-methyladenosine RNA methylation, the techniques for detecting *N* ^6^-methyladenosine methylation, the role of *N* ^6^-methyladenosine modification in cancer and other diseases, and the potential clinical applications.

## 1 Introduction

With the development of epigenetics, epitranscriptomics has emerged as another level of epigenetic regulation and has recently become a research hotspot. The epitranscriptome refers to the relevant functional changes of the transcriptome without any alteration of the RNA sequence. Conceptually, the epitranscriptome covers all the chemical modifications of RNA dynamically regulated by the removal and addition of various chemical groups in cells (Saletore et al., 2012). To date, over 170 RNA chemical modifications have been identified, including *N* ^6^-methyladenosine (m^6^A), *N* ^1^-methyladenosine (m^1^A), 5-hydroxymethylcytosine (hm^5^C), 5-methylcytidine (m^5^C), ribose 2′-O-methylation (Nm), 1-methylguanine (m^1^G), 6-methylguanine (m^6^G), 7-methylguanine (m^7^G), *N* ^4^-acetylcytidine (ac^4^C) and pseudouridine (w) (Wiener and Schwartz, 2021), but most of their functions are largely unknown. Among them, 72 variants of methyl group modifications are conjugated at distinct positions in RNA bases. Since the first discovery of m^6^A RNA methylation in 1974, it has been identified as one of the most prevalent and abundant posttranscriptional modifications, widely distributed in many biological species, such as mammals (Desrosiers et al., 1974; Liu et al., 2022), plants (Yue et al., 2019; Yu Q. et al., 2021), zebrafish (Zhao et al., 2017), insects (Yang et al., 2021), yeast (Yadav and Rajasekharan, 2018), bacteria (Deng et al., 2015) and viruses (Bayoumi et al., 2020), accounting for approximately 50% of total methylated ribonucleotides in total RNA content (Wei et al., 1975). It is estimated that more than 7,000 mRNAs with m^6^A modification are distributed in mammalian cells, with a frequency of 0.1–0.6% of adenosines (Ke et al., 2015). In addition, m^6^A deposition also exists in other types of RNA, including rRNA, tRNA, small nuclear RNA (snRNA), small nucleolar RNA (snoRNA), long noncoding RNA (lncRNA), microRNA (miRNA), and circular RNA (circRNA) (Pendleton et al., 2017; van Tran et al., 2019; Dai et al., 2020).

**FIGURE 1:**
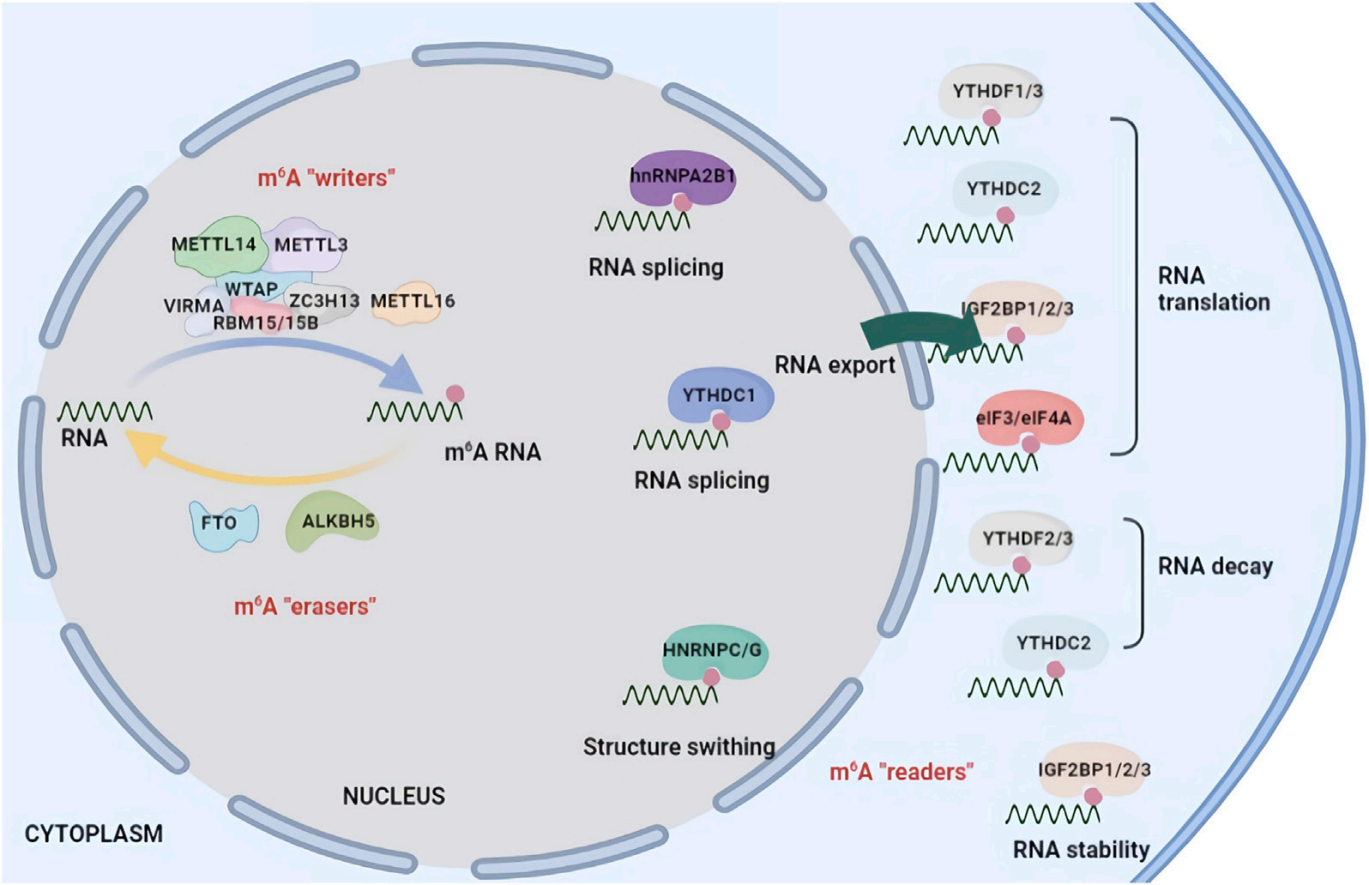
Chemical basis and molecular functions of m^6^A machinery. m^6^A methylation is catalysed by a multicomponent m^6^A methyltransferase complex (“m^6^A writers”) that is composed of two predominant proteins, METTL3 and METTL14, as well as their cofactors WTAP, HAKAI, RBM15, RBM15B, VIRMA and ZC3H13. m^6^A methylation can be removed by demethylases (“m^6^A erasers”) including FTO and ALKBH5. m^6^A modification affects RNA fate by recruiting m^6^A-binding proteins (“m^6^A readers”) such as YTHDF1/2/3, YTHDC1/2, IGF2BP1/2/3 and HNRNPC/A2B1. m^6^A methylation is involved in RNA splicing, localization, translation efficiency, stability and degradation.

In 2012, several decades after the first discovery of m^6^A RNA methylation, utilizing m^6^A -specific antibodies, two groups independently conducted fragmented RNA immunoprecipitation and subsequent high-throughput RNA deep sequencing (termed “MeRIP-seq” or “m^6^A-seq”) to map m^6^A throughout the transcriptome in humans and mice (Dominissini et al., 2012; Meyer et al., 2012). The results first revealed that m^6^A modification was widely distributed in mRNA, additionally, m^6^A modification mainly occurred in the common motif RRACH (R = G or A, H = A, C or U), but only 1–5% of these sites were methylated in cellular RNA. Notably, most m^6^A peaks were evolutionarily conserved between the human and mouse transcriptomes. More surprisingly, m^6^A modification on the RRACH motif was preferentially enriched in 3′-untranslated regions (3′-UTRs) and near stop codons of coding sequences (CDS) (Meyer et al., 2012), indicating that the RRACH motif is not sufficient for the determination of m^6^A modification. The m^6^A levels vary in distinct cell contexts and are involved in multiple RNA metabolism pathways, such as splicing, localization, translation efficiency, stability and degradation (Kasowitz et al., 2018; Liu et al., 2020; He and He, 2021), ultimately affecting various physiological and pathological processes.

m^6^A RNA methylation is dynamic and reversible and is also tightly regulated by three types of proteins, methyltransferases (“writers”), demethylases (“erasers”) and m^6^A binding proteins (“readers”). The m^6^A methylase complex was first purified in the 1990s (Bokar et al., 1994). METTL3 is identified as a predominant component and contains a catalytically active subunit. Another methyltransferase, METTL14, is essential for structural stability to facilitate the catalysis of m^6^A methylation. The larger methyltransferase holocomplex is composed of WTAP, HAKAI, RBM15, RBM15B, VIRMA and ZC3H13 and is approximately 1,000 kDa in size (Oerum et al., 2021). FTO was the first m^6^A demethylase identified in 2011, followed by ALKBH5 (Niu et al., 2013). These two enzymes can remove m^6^A methylation from RNA, posing a novel research field of regulation for epitranscriptomics. The functions of m^6^A RNA methylation are mediated by different m^6^A “readers” that selectively recognize m^6^A in a direct or indirect manner and conduct distinct functions (Shi et al., 2019). The m^6^A binding proteins include the YTH family, the heterogeneous nuclear ribonuclease (HNRNP) family and FMRP (Figure 1).

In this review, we will address the regulatory mechanisms of m^6^A RNA methylation, the techniques for detecting m^6^A methylation, the role of m^6^A modification in cancer as well as the potential clinical applications.

## 2 Regulation of *N* ^6^-methyladenosine RNA modification

**FIGURE 2:**
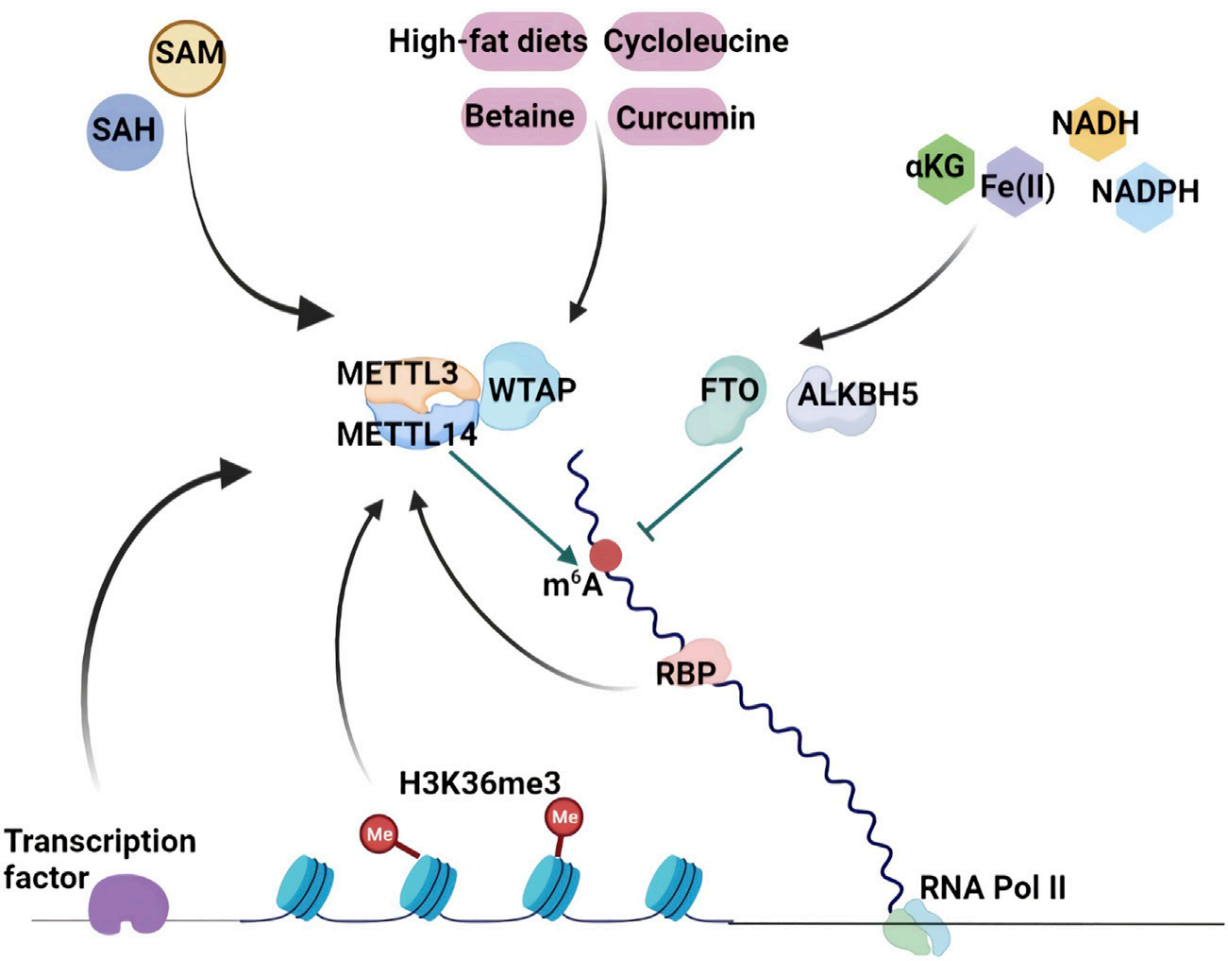
The biogenesis and regulatory mechanisms of RNA m^6^A Methylation. Methyltransferases and demethylases dynamically and reversibly direct the addition and removal of m^6^A RNA methylation. H3K36me3 guides m^6^A deposition in stop codons of CDSs and 3′-UTRs. The dynamics of m^6^A regulation can be achieved by some transcription factors that recruit the m^6^A methyltransferase complex to specific RNA loci in distinct cellular contexts. In addition, m^6^A methylation patterns are modulated by nutrition and metabolites including SAM, SAH, cycloleucine, betaine, curcumin, high-fat diets, αKG, NADH and NADPH.

m^6^A RNA methylation is functionally important and tightly modulated by several molecular mechanisms in eukaryotes. As described above, catalytic enzymes, methyltransferases and demethylases dynamically and reversibly direct the addition and removal of m^6^A RNA methylation, and are termed “m^6^A writers” and “m^6^A erasers”, respectively. Some binding proteins (“m^6^A readers”) recognize and function by decoding m^6^A methylation as well as recruiting downstream functional protein complexes to mediate biological activities. Histone modification also guides m^6^A deposition in stop codons of CDSs and 3′-UTRs. The dynamics of m^6^A regulation can be achieved by some transcription factors that recruit the m^6^A methyltransferase complex to specific RNA loci in distinct cellular contexts. In addition, nutrition and metabolites can reverse and modulate m^6^A methylation patterns (Figure 2).

### 2.1 RNA *N* ^6^-methyladenosine machinery

m^6^A methylation is catalysed by a multicomponent m^6^A methyltransferase complex that is composed of two predominant proteins, methyltransferase-like 3 (METTL3) and methyltransferase-like 14 (METTL14), and their cofactors WTAP, HAKAI, RBM15, RBM15B, VIRMA and ZC3H13(Knuckles et al., 2018; Yue et al., 2018; Bawankar et al., 2021). Although both METTL3 and METTL14 have methyltransferase domains, only METTL3 contains a catalytically active subunit, which requires S-adenosylmethionine (SAM) as a substrate to mediate catalytic activity. The SAM binding pocket is distributed on one side of the central *ß*-sheet and is enclosed by the catalytic site loop (Wang et al., 2016). METTL14 is associated with the stabilization of the conformation between METTL3 and the RNA substrate (Zhou H. et al., 2021). The METTL3-METTL14 complex is formed in the cytoplasm and is located in the nucleus, and it induces m^6^A methylation (Scholler et al., 2018). Both *in vitro* methylation assays and CLIP combined with photoactivatable ribonucleoside-enhanced crosslinking (PAR-CLIP) suggested that the METTL3-METTL14 complex efficiently catalyses m^6^A methylation on the GGACU or GGAC motifs of RNAs, consistent with the RRACH motif of a previous study (Liu et al., 2014). The depletion of *Mettl3* and/or *Mettl14* could greatly reduce the peak numbers and the enrichment of m^6^A in the global transcriptome (Vu et al., 2017; Weng et al., 2018). Except for these two modulators, other m^6^A writers lack methyltransferase activity. WTAP guides METTL3 and METTL14 into nuclear speckles to efficiently methylate target RNAs (Ping et al., 2014). RBM15 and RBM15B have been confirmed to interact with WTAP by coimmunoprecipitation and bind to specific RNA regions that are adjacent to the DRACH sequence, suggesting that RBM15 and RBM15B can recruit the METTL3-WTAP complex and direct these methyltransferases to DRACH consensus sequence sites for m^6^A modification (Patil et al., 2016; Shi et al., 2019). RBM15 and RBM15B targets induce X-chromosome inactivation and gene silencing by binding to lncRNA XIST, and VIRMA prefers to mediate alternative polyadenylation and mRNA methylation near the 3′-UTRs and stop codon regions (Yue et al., 2018; Zhu et al., 2021). ZC3H13 complexes with WTAP or other cofactors to regulate nuclear m^6^A RNA methylation (Wen et al., 2018). Recently, METTL16, as an independent RNA methyltransferase, was shown to catalyse the m^6^A methylation of U6 spliceosomal RNA (snRNA) and U6-like hairpins of *Mat2a* mRNA (Shima et al., 2017). ZCCHC4, a new m^6^A methyltransferase, was found to specifically recognize the AAC motif associated with rRNA methylation (Ma et al., 2019).

After deposition, m^6^A methylation is reversible and can be removed by demethylases (“m^6^A erasers”). FTO belongs to the nonheme Fe(II)- and α-KG-dependent dioxygenase AlkB family. FTO was the first enzyme reported to modulate m^6^A demethylation in 2011. In addition, m^6^Am RNA, m^1^A RNA, m^3^T single-stranded DNA and m^3^U single-stranded RNA modifications can be demethylated by FTO (Wei et al., 2018). Mauer et al. found that the catalytic activity of FTO towards m^6^Am was approximately 10 times greater than that towards m^6^A (Mauer et al., 2017). It has been reported that snRNA and snoRNA are targets of FTO (Mauer et al., 2019). Another m^6^A demethylase, ALKBH5, is a member of the ALKB family and seems to be specific for m^6^A RNA methylation (Yu F. et al., 2021).

m^6^A modification affects RNA fate by recruiting m^6^A-binding proteins (m^6^A ‘readers’) such as YTH domain-containing proteins, insulin-like growth factor 2 mRNA-binding proteins IGF2BP1-3 and the heterogeneous nuclear ribonuclease (HNRNP) family (Zhao et al., 2020). In mammals, YTH domain-containing proteins contain five members: YTHDC1, YTHDC2, YTHDF1, YTHDF2, and YTHDF3. YTHDC1 play roles in alternative splicing events, nuclear export of RNAs into the cytoplasm and mRNA decay (Xiao et al., 2016). YTHDC2 promotes target mRNA translation (Mao et al., 2019). YTHDF1 interacts with the translation initiation factors eIF3 and eIF4A3 to enhance the translation efficiency of m^6^A-modified mRNAs (Liu et al., 2020; Cai et al., 2022). YTHDF2 and YTHDF3 are involved in the degradation of target mRNAs associated with p-bodies, and the depletion of *Ythdf2* and *Ythdf3* causes a considerable increase in m^6^A mRNA abundance in cells. hnRNPA2B1, hnRNPC and hnRNPG are related to mRNA splicing (Bi et al., 2019). In contrast, IGF2BP1/2/3, FMRP, and PRRC2A are essential for the stabilization of m^6^A-modified transcripts in a m^6^A-dependent manner (Huang et al., 2018; Edens et al., 2019; Wu et al., 2019).

### 2.2 Histone modification guides *N* ^6^-methyladenosine deposition

Histone modification has an effect on m^6^A deposition. H3 lysine 36 trimethylation (H3K36me3) is a classical transcription activator that shows a similar m^6^A distribution. H3K36me3 chromatin immunoprecipitation (ChIP)-seq analysis indicated that approximately 70% of H3K36me3 sites overlapped with m^6^A peaks, suggesting a close connection between H3K36me3 and m^6^A modification (Huang et al., 2019) (Figure 2). SETD2 and KDM4A are H3K36me3 methyltransferase and H3K36me3 demethylase, respectively (Mar et al., 2017). More surprisingly, changes in H3K36me3 levels by utilizing dCas9-SETD2 or dCas9-KDM4A can significantly alter m^6^A abundance in human and mouse transcriptomes, revealing that H3K36me3 is a modulator of m^6^A deposition. Of note, most H3K36me3-dependent m^6^A sites are targeted by METTL3, METTL14 and WTAP, demonstrating the association between H3K36me3 and m^6^A modification. In addition, METTL14 can directly bind to H3K36me3, which leads to the recruitment of other m^6^A methyltransferases to activate RNA Pol II and controls m^6^A methylation on mRNA (Huang et al., 2019; Zhou X. L. et al., 2021). Therefore, the relationship between H3K36me3 and m^6^A provides a new way to enrich multiple aspects of gene expression regulation.

### 2.3 Transcription factors affect *N* ^6^-methyladenosine deposition

Transcription factors also recruit m^6^A methyltransferases to regulate RNA modification in specific cell contexts. Zinc-finger protein 217 (ZFP217) is a transcriptional activator of some key pluripotency genes that are essential for maintaining self-renewal in mESCs. METTL3 can be bound and sequestered by ZFP217, preventing the formation of the m^6^A methyltransferase complex and m^6^A methylation on ZFP217 target transcripts (Aguilo et al., 2015). In contrast, the transcription factors SMAD2 and SMAD3 preferentially recruit the METTL3-METTL14-WTAP methyltransferase complex to their target transcripts and increase the m^6^A modification of target transcripts (Bertero et al., 2018). Different functions of m^6^A deposition on the target transcripts determine the distinct roles of ZFP217 and SMAD2/3 in ESCs. Another study reported that METTL3 could interact with the CAATT-box binding protein CEBPZ on target transcripts and mediate m^6^A modification to promote the translation of target mRNAs that maintain the leukaemic state in acute myeloid leukaemia (AML) cells (Barbieri et al., 2017). Frequently, these transcription factors manipulate m^6^A deposition on a subset of target transcripts in specific cellular contexts to implement dynamic regulation of gene expression (Figure 2).

### 2.4 Nutritional metabolism and metabolites regulate *N* ^6^-methyladenosine deposition

Evidence has shown that nutritional challenge and metabolites play crucial roles in the manipulation of m^6^A deposition. Cycloleucine is a competitive inhibitor of methionine adenosyltransferase that decreases m^6^A RNA methylation levels by reducing SAM concentrations (Kang et al., 2018). Betaine, as a methyl donor for SAM synthesis, prompts m^6^A methylation by suppressing FTO expression in the adipose tissues of high-fat diet-fed mice, which increases the expression of the mitochondrial protein PGC-1α to improve metabolic disorder (Zhou et al., 2015). It has been reported that curcumin enhances m^6^A modification by decreasing ALKBH5 and increasing METTL3 and METTL14 expression in the livers of piglets (Ding et al., 2016). Undoubtedly, high-fat diets affect m^6^A modification in various models and tissues (Tung et al., 2010; Wu et al., 2020) (Figure 2).

SAM, a common methyl donor, is involved in most cellular methylation processes. The change in cellular SAM concentration affects DNA and histone methylation as well as RNA methylation (Duncan et al., 2013). METTL3 requires SAM as a substrate to mediate catalytic activity and m^6^A writing. Interestingly, the SAM binding affinity of METTL3 is regulated by substrate RNA availability. S-adenosyl homocysteine (SAH) is the metabolite of SAM during the methylation reaction that can strongly inhibit METTL3 methyltransferase activity (Li F. et al., 2016; Selberg et al., 2019). Demethylases, FTO and ALKBH5 are 2-oxoglutarate (αKG)- and Fe(II) dependent. The FTO and ALKBH5 mutants of the αKG-Fe(II) oxygenase domain lost the catalytic activities of m^6^A demethylation (Feng et al., 2014; Zhang X. et al., 2019). Recently, NADH and NADPH were identified as the direct binding partners of FTO by using a florescence quenching assay. Both NADH and NADPH could enhance FTO demethylase activity, indicating that reducing NADPH and NADH may attenuate demethylation reactions. Conversely, the induction of NADPH by glucose injection or a high-fat diet suppressed m^6^A modification (Figure 2). In contrast, the depletion of G6P dehydrogenase (G6PD) or NAD kinase (NADK) enhanced cellular m^6^A abundance, which was reversed by NADPH supplementation (Wang L. et al., 2020).

## 3 Approaches for detecting *N* ^6^-methyladenosine RNA methylation

Several techniques have been developed for detecting m^6^A RNA methylation (Table 1). Although immuno-northern blot and m^6^A dot blot facilitate easier and faster observation of global m^6^A levels, the disadvantages are obvious with lower sensitivity and are semiquantitative accuracy (Nagarajan et al., 2019). High-performance liquid chromatography‒mass spectrometry (HPLC‒MS/MS) is used for quantifying m^6^A levels with high sensitivity; however, this approach cannot provide details about RNA sequence and localization information (Thuring et al., 2017). Site-specific cleavage and radioactive labelling followed by ligation-assisted extraction and thin-layer chromatography (SCARLET) is suitable for stoichiometric quantification, but it is very tedious and is only used to validate the known m^6^A changes at a given site (Liu and Pan, 2016). Above all, these techniques are not appropriate for widespread identification and localization of modified sites; subsequently, high-throughput sequencing methods have emerged and have been rapidly developed. m^6^A-specific antibody-based high-throughput sequencing strategies are widely used for the identification of m6A, which include m^6^A-Seq, MeRIPSeq, PA-m^6^A-Seq, m^6^A-CLIP and m^6^A individual-nucleotide resolution cross-linking and immunoprecipitation (miCLIP) (Dominissini et al., 2012; Meyer et al., 2012; Chen et al., 2015; Ke et al., 2015; Linder et al., 2015). MeRIP-seq was first identified in 2012, allowing for m^6^A analysis with 100- to 200-nucleotide resolution. In terms of MeRIP-seq, mRNA was fragmented into 100-nucleotide lengths and immunoprecipitated by a m^6^A-specific antibody with the combination of high-throughput deep sequencing. miCLIP enables the detection of m^6^A residues at precise positions with single-nucleotide resolution. However, the number of identified m^6^A peaks is limited due to the low cross-linking efficiency of this method. m^6^A-specific antibody-based sequencing approaches have some obvious drawbacks. m^6^A antibodies are not strikingly specific for m^6^A, may also bind to m^6^Am sites and other non-m^6^A-specific sequences. Distinct commercial m^6^A antibodies show differences in affinity for m^6^A (Haussmann et al., 2016). Endoribonuclease-based techniques include antibody-free m^6^A sequencing methods such as m^6^A-REF-Seq and MAZTER-Seq and rely on the endoribonuclease activity of MazF (Zhang Z. et al., 2019; Garcia-Campos et al., 2019). Therefore, the motif preference of endoribonuclease determines the limitation, and these methods detect a portion of the m^6^A sites. Recently, chemical labelling strategies have been reported, including m^6^A-SEAL, m^6^A-label-seq and m^6^A-SAC-seq (Wang Y. et al., 2020; Shu et al., 2020; Hu et al., 2022). Nevertheless, improvements in the chemical labelling efficiency are needed. A specific method for m^6^A detection in the global transcriptome should be validated by other techniques to obtain a more accurate m^6^A landscape.

**TABLE 1:** Approaches for the detection of m^6^A RNA methylation.

Approaches	Principle	Advantages	Limitations	References
m^6^A dot blot	Antibody based immunoblot	Easy, fast	Low sensitive, semi-quantitative	Nagarajan et al. (2019)
HPLC-MS/MS	Mass spectrum	High sensitive, quantitative	Lack of RNA sequence and localization	Thuring et al. (2017)
SCARLET	Thin-layer chromatography	Quantitative	Complicated, low-throughput	Liu and Pan, (2016)
m^6^A-Seq	Antibody based sequencing	High-throughput	100–200 nucleotide resolution	Dominissini et al. (2012)
MeRIPSeq	Antibody based sequencing	High-throughput	100–200 nucleotide resolution, antibody specificity	Meyer et al. (2012)
miCLIP	Antibody based sequencing	High-throughput, single site	Low cross-linking efficiency, antibody specificity	Linder et al. (2015)
MAZTER-Seq	Endoribonuclease based sequencing	High-throughput, single site	Preference of the enzyme	Garcia-Campos et al. (2019)
m^6^A-REF-Seq	Endoribonuclease based sequencing	High-throughput, single site	Preference of the enzyme	Zhang Z. et al. (2019)
m^6^A-SEAL	Chemical labeling sequencing	High-throughput, single site	Chemical labeling efficiency	Wang Y. et al. (2020)
m^6^A-label-seq	Chemical labeling sequencing	High-throughput, single site	Chemical labeling efficiency	Shu et al. (2020)
m^6^A-SAC-seq	Chemical labeling sequencing	High-throughput, single site	Chemical labeling efficiency	Hu et al. (2022)

## 4 Role of *N* ^6^-methyladenosine RNA methylation in human diseases

**FIGURE 3:**
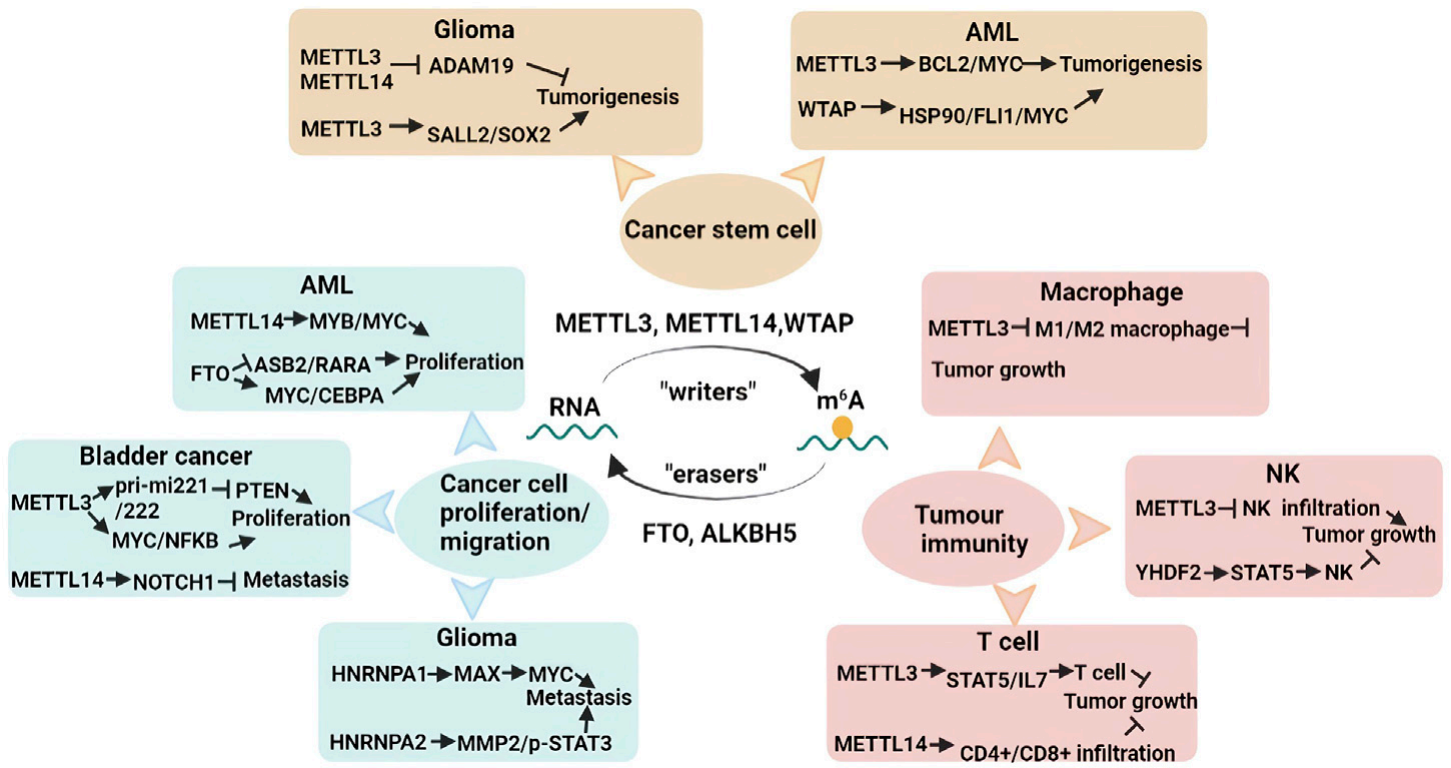
Diverse functions of m^6^A RNA methylation in cancers. m^6^A RNA methylation plays a crucial role in tumorigenesis and cancer progression. m^6^A RNA methylation is reversible and is also tightly regulated by “writers” and “readers”. m^6^A RNA methylation contributes to mediating CSC pluripotency, cancer proliferation, tumour metastasis, and tumor immunity. More details are shown in a graphical form.

m^6^A RNA methylation is involved in multiple RNA metabolism pathways, such as splicing, localization, translation efficiency, stability and degradation, ultimately affecting various physiological and pathological processes. For instance, m^6^A methylation has been demonstrated to regulate the haematopoietic system, the central nervous system, immunity stemness, mammalian spermatogenesis and brain development. The dysregulation of m^6^A methylation is associated with various diseases, especially cancer (Figure 3).

### 4.1 *N* ^6^-methyladenosine and cancer

#### 4.1.1 *N* ^6^-methyladenosine in cancer stem cells

Cancer stem cells (CSCs) are a type of cells possessing a stem cell-like capacity to self-renew, differentiate and survive and give rise to many types of cancers (Nassar and Blanpain, 2016). CSCs lead to the tolerance of standard therapeutics and CSCs, tumour recurrence, and distant metastasis (Walcher et al., 2020). Cui et al. found that m^6^A methylation played a role in the tumorigenesis of glioma stem cells (GSCs). *Mettl3/14* knockdown prominently promoted GSC self-renewal and tumorigenesis by decreasing m^6^A levels, whereas METTL3 overexpression exerted negative effects (Cui et al., 2017; Zhang et al., 2017). MeRIP-seq revealed that silencing *Mettl3/14* altered m^6^A enrichment and that m^6^A RNA methylation of ADAM19 regulated GSC self-renewal. Similarly, Visvanathan et al. confirmed that METTL3 was essential for glioma cell differentiation and GSC maintenance. Furthermore, METTL3 is also involved in radiosensitivity and DNA repair through the SOX2 axis in GSCs (Visvanathan et al., 2018).

Wang et al. provided the first evidence of the interplay between m^6^A methylation and osteosarcoma stem cells (OSCs). Compared with non-OSCs, METTL14 and FTO were significantly reduced in OSCs. Meanwhile, MeRIP-seq and RNA-seq analyses of OSCs and non-OSCs revealed that differentially expressed genes containing differentially methylated m^6^A peaks were associated with the Wnt pathway and the pluripotency of stem cells (Wang et al., 2019).

Ly P Vu and others revealed that METTL3 expression was elevated in leukaemia cells compared with normal haematopoietic cells. miCLIP analysis showed that METTL3 enhanced the m^6^A methylation of target genes such as *Bcl2*, *Myc* and *Pten* in the human acute myeloid leukaemia (AML) MOLM-13 cell line, which promoted the mRNA translation of these genes, thereby retaining pluripotency properties and inhibiting cell differentiation. Notably, silencing of *Mettl3* in human myeloid leukemia cell lines promoted cell differentiation and cell apoptosis (Vu et al., 2017). WTAP is associated with haematopoietic stem cell (HSC) homeostasis and haematopoietic regeneration (Wang H. et al., 2018; Li et al., 2018). Recent research conducted by Bansal et al. suggested a role for m^6^A modification in myeloid leukaemia. WTAP expression was increased in AML cells derived from patients with AML, while knockdown of *Wtap* led to the repression of cell proliferation, the activation of cell differentiation and apoptosis in a leukaemia cell line (Bansal et al., 2014) (Figure 3).

#### 4.1.2 *N* ^6^-methyladenosine in cancer cell proliferation and migration

Numerous reports have elucidated that m^6^A modification is involved in cancer cell proliferation and tumour metastasis in different types of cancers. Recent findings revealed that METTL14 exerted an oncogenic function by increasing the expression levels of targets such as MYB and MYC in AML, while SPI downregulated the expression level of MEETL14 (Weng et al., 2018). FTO demethylase is elevated and plays an oncogenic role in AML. It has been demonstrated that a high level of FTO prompts cell proliferation and viability, whereas it reduces cell apoptosis and global m^6^A methylation by repressing the expression of ASB2 and RARA (Li Z. et al., 2017). Su et al. observed that R-2-hydroxyglutarate (R-2HG) attenuated FTO activity and augmented global m^6^A modification in R-2HG-sensitive AML cells, which decreased the stability of *Cebpa*/*Myc* mRNA and the activities of relevant cell signalling pathways (Su et al., 2018).

In bladder cancer, METTL3 interacts with DGCR8 to facilitate pri-miR221/222 maturation, which leads to a decrease in PTEN and ultimately promotes cell proliferation (Han et al., 2019). Similarly, another study showed that METTL3 was significantly elevated in bladder cancer, and *METTL3* knockdown dramatically suppressed cancer cell proliferation, cell invasion, and tumour formation through the AFF4/MYC/NF-kB axis cell signalling pathway (Cheng et al., 2019). Xie et al. revealed that METTL3 also binds to YTHDF2, which induces the degradation of target tumour suppressor mRNAs, including *Klf4* and *Setd7*, regulating the progression of bladder cancer (Xie et al., 2020). Nevertheless, METTL14 has been confirmed to be decreased in bladder cancer, and depletion of *Mettl14* accelerated cell proliferation, tumour metastasis and self-renewal by decreasing the stability of m^6^A-modified Notch1 transcripts (Gu et al., 2019).

Evidence indicates that the hnRNPA1 expression level is elevated by EGFRvIII, leading to increased glycolytic gene expression in gliomas. Meanwhile, hnRNPA1 promotes *Max* mRNA splicing and then induces the generation of Delta Max, which promotes Myc-dependent cell transformation (Babic et al., 2013). Another study showed that silencing *hnRNPA2* represses cancerous cell viability, cell invasion, tumour metastasis and chemoresistance by decreasing the expression of MMP-2 and phospho-STAT3. Notably, hnRNPA2 has been regarded as an oncogenic driver in gliomas (Deng et al., 2016) (Figure 3). Numerous studies have described how m^6^A methylation contributes to cell proliferation, cell invasion, and tumour metastasis in other cancers, including breast cancer, ovarian cancer, cervical cancer, prostate cancer, lung cancer, hepatocellular carcinoma, gastric carcinoma, pancreatic cancer and colorectal cancer (Hu et al., 2019; Yang et al., 2019; Ma et al., 2020; Yang et al., 2020; Guo et al., 2021; Zhang and Xu, Forthcoming 2022).

#### 4.1.3 *N* ^6^-methyladenosine in tumour immunity

Recent studies have revealed that m^6^A RNA methylation induces the activation and infiltration of various immune cells into the tumour microenvironment (TME), influencing the efficacy of cancer immunotherapy. Macrophages are closely associated with tumour initiation and progression. Yin et al. elucidated that METTL3 in macrophages regulates tumour development. Silencing of *Mettl3* in macrophages facilitated tumour growth and lung metastasis. The TME was reshaped by inducing regulatory T (Treg) cells into tumour sites and promoting the infiltration of M1-and M2-like tumour-associated macrophages (Yin et al., 2021). *Mettl14* knockdown in macrophages suppressed the antitumour activity of CD8^+^ T cells and improved tumour growth (Dong et al., 2021).

Natural killer (NK) cells play an important role in cancer immune surveillance and can directly recognize and kill cancer cells. YTHDF2 was critical for modulating NK-cell maturation, NK-cell homeostasis, IL-15-mediated survival, and antitumor activity due to the regulation of downstream target genes such as *Stat5*, *Eomes* and *Tardbp* (Ma et al., 2021). Song et al. found that METTL3 expression was decreased in tumour-infiltrating NK cells of cancer patients. In mice, they observed that depletion of *Mettl3* enhanced NK-cell responsiveness to IL-15 and promoted tumour progression and metastasis by targeting SHP-2 (Song et al., 2021).

Silencing of *Mettl3* in CD4^+^ T cells destroyed T-cell differentiation and homeostasis by repressing the activation of IL-7-mediated STAT5/suppressor of cytokine signalling (Li H. B. et al., 2017). Yao et al. revealed that conditional depletion of *Mettl3* in CD4^+^ T cells inhibited T follicular helper differentiation and maturation, thereby preventing the antibody response of B cells by promoting the degradation of m^6^A-modified *Tcf7* mRNA (Yao et al., 2021). In breast cancer, the expression levels of METTL14 have a positive correlation with the infiltration of CD4^+^ T cells, CD8^+^ T cells, dendritic cells, macrophages and neutrophils, but they negatively correlated with Treg cells in breast cancer (Gong et al., 2020) (Figure 3).

### 4.2 *N* ^6^-methyladenosine and other human diseases

Emerging evidence has demonstrated that m^6^A RNA methylation is closely related to other human diseases, including cardiovascular disease, metabolic syndrome, psychiatric disorders and autoinflammatory disorders. Dorn et al. (Dorn et al., 2019) showed that the global m^6^A level of cardiomyocytes was significantly elevated in response to hypertrophic stimulation and that METTL3 played a vital role in cardiomyocyte hypertrophy. Notably, upregulated m^6^A modification resulted in compensated cardiac hypertrophy, whereas downregulated m^6^A levels led to eccentric cardiomyocyte remodelling and dysfunction. Overexpression of *Mettl3* increased the expression levels of mitogen-activated protein (MAP)3K6, MAP4K5, and MAPK14 in cardiomyocytes, which was positively correlated with cardiomyocyte size, revealing that METTL3 is sufficient to drive cardiomyocyte hypertrophy. However, no histopathologic changes were observed in *Mettl3*-overexpressing mice.

Zhou et al. identified that YTHDC2 was significantly repressed in nonalcoholic fatty liver disease (NAFLD) patients, and *Ythdc2*-depleted hepatocytes led to the accumulation of excessive triglycerides (TGs) by reducing the expression levels of lipogenic genes, including fatty acid synthase, sterol regulatory element-binding protein 1c, and acetyl-CoA carboxylase 1 (Zhou B. et al., 2021). Furthermore, m^6^A sequencing was performed in human type 2 diabetes islets, and sequencing analysis showed that multiple hypomethylated transcripts were associated with insulin secretion, the insulin/IGF1 signalling pathway and cell cycle progression. *Mettl14* knockout in mouse ß-cells caused a reduction in global m^6^A levels, giving rise to a similar islet phenotype in human T2D (De Jesus et al., 2019).

In Alzheimer’s disease mouse models, the global m^6^A level was increased in the hippocampus and the cortex compared to C57BL/6 mice, and the interaction of FTO and APOE contributed to the increase in Alzheimer’s disease risk. The overall m^6^A level was elevated in the cortex and the hippocampus of APP/PS1 (Alzheimer’s disease) mice compared to C57BL/6 control mice, and FTO was found to interact with APOE, which was associated with Alzheimer’s disease risk in a prospective cohort study (Keller et al., 2011; Han et al., 2020). Huang et al. found that ALKBH5/FAAH enhanced the expression of circSTAG1, which attenuated astrocyte dysfunction and depressive-like behaviours *in vitro* and *in vivo* (Huang et al., 2020).

m^6^A methylation also has a contributory effect on autoinflammatory disorders. Luo et al. identified that the expression levels of METTL14, ALKBH5 and YTHDF2 were downregulated in peripheral blood mononuclear cells of systemic lupus erythematosus (SLE) patients (Luo et al., 2020). In addition, multivariate logistic regression analysis showed that repression of ALKBH5 and YTHDF2 was considered a risk factor for SLE. However, direct mechanistic data should be provided for the function of m^6^A modification in SLE progression. Another study found that *Mettl14* knockdown inhibited the activation of Treg cells, which impaired the balance between Th17 and Treg cells, leading to the development of spontaneous colitis (Lu et al., 2020).

## 5 Potential clinical applications of *N* ^6^-methyladenosine RNA modification

Due to the major role of m^6^A RNA modification in tumour and other disease progression, m^6^A-associated proteins can be developed as potential therapeutic targets for tumours and other diseases (Table 2). METTL3 attenuates the sensitivity of colon cancer cells to chemotherapy of L-OHP and CPT-11 by upregulating the expression level of CBX8 in a m^6^A-dependent manner (Zhang Y. et al., 2019). Meanwhile, *Ythdf1*-depleted colon cancer cells are more sensitive to 5-FU and L-OHP (Nishizawa et al., 2018). Another study identified that gemcitabine drove the apoptosis of pancreatic cancer cells with low METTL3 expression (Taketo et al., 2018). The combination of m^6^A methylation and chemotherapeutic drugs contributes to resolving drug resistance in tumours. Cas13-directed methyltransferase has been used for cancer treatment, targeting m^6^A of specific RNA loci (Wilson et al., 2020; Lo et al., 2022).

**TABLE 2:** Overview of the small-molecule drugs and m^6^A-related factor inhibitors described in the text.

Drug/inhibitor	Molecular structure	m^6^A proteins involved	Function	References
SAM mimic	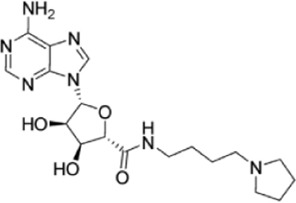	METTL3	METTL3 inhibitor	Bedi et al. (2020)
UZH1a	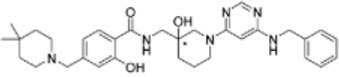	METTL3	METTL3 inhibitor	Moroz-Omori et al. (2021)
Rhein	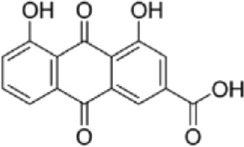	FTO	FTO inhibitor	Li Q. et al. (2016)
Fluorescein derivative	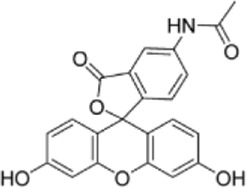	FTO	FTO inhibitor	Wang et al. (2015)
Radicicol	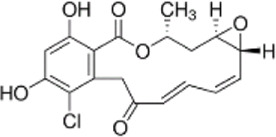	FTO	FTO inhibitor	Wang R. et al. (2018)
Meclofenamic acid (MA)	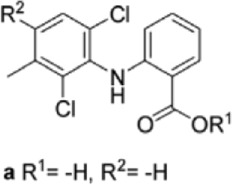	FTO	FTO inhibitor	Huang et al. (2015)
CHTB	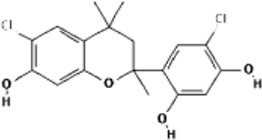	FTO	FTO inhibitor	Qiao et al. (2016)
Entacapone	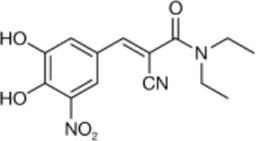	FTO	FTO inhibitor	Peng et al. (2019)
N-CDPCB	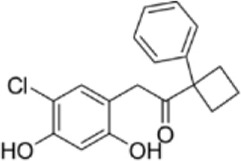	FTO	FTO inhibitor	Qiao et al. (2017)
Dac51	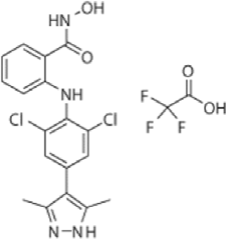	FTO	FTO inhibitor	Liu et al. (2021)
MV1035	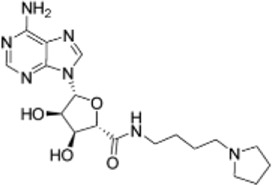	ALKBH5	ALKBH5 inhibitor	Malacrida et al. (2020)

Specific inhibitors based on m^6^A-related enzymes have been studied. Bedi et al. (Bedi et al., 2020) performed a virtual screening assay to identify potential METTL3 inhibitors from 4,000 adenosine derivatives. One compound, a SAM mimic, was found to be the first inhibitor of METTL3. However, the therapeutic value of this compound is still somewhat limited due to cell penetration issues and nonspecific targets. They also identified another METTL3 inhibitor, UZH1a, that decreased the m^6^A/A ratios in three different cell lines, MOLM-13, HEK293T and U2OS, but the specificity of UZH1a needs further improvement (Moroz-Omori et al., 2021).

Currently, more papers are focused on FTO inhibitors. Rhein, a competitive inhibitor of FTO, binds to the catalytic domain and blocks the catalytic activity of FTO (Li Q. et al., 2016). Fluorescein derivatives, radicicol, IOX3 and MA (a nonsteroidal anti-inflammatory drug) were subsequently identified to repress FTO expression (Huang et al., 2015; Wang et al., 2015; Wang R. et al., 2018). Similar to MA, MA2, an isomer of MA, interacts with FTO to increase the m^6^A level but possesses better cell penetration ability. In addition, CHTB, entacapone and N-CDPCB also repress the demethylase activity of FTO (Qiao et al., 2016; Qiao et al., 2017; Peng et al., 2019). Dac51 inhibits the activity of FTO, promotes T cell response and enhances the anti-PD-1 therapy (Liu et al., 2021). Small-molecule FTO inhibitors have been developed as potential drugs not only for tumour therapy but also for the treatment of neurological diseases, cardiovascular disease, metabolic syndrome and autoinflammatory disorders. To date, little is known about specific ALKBH5 inhibitors. However, the imidazobenzoxazin-5-thione MV1035 was found to be a potential candidate ALKBH5 inhibitor (Malacrida et al., 2020). ALK-04 as an inhibitor of ALKBH5 reduces the infiltration of myeloid-derived suppressor cells and Treg cells and suppresses tumour growth by enhancing the therapeutic effect of anti-PD-1 treatment (Li et al., 2020). (Table 2).

## 6 Conclusions and future prospects

Despite the initial discovery of m^6^A in 1974, m^6^A modification has received much less attention than histone and DNA epigenetic modifications. Since the establishment of MeRIP-Seq for mapping m^6^A deposition at a transcriptome-wide level in 2012, interest in studying m^6^A methylation has grown rapidly. The level of m^6^A is dynamically and reversibly regulated by various effectors termed “writers”, “erasers” and “readers”. The METTL3-14-WTAP methyltransferase complex is the core constituent of most m^6^A writers, whereas FTO and ALKBH5, as m^6^A demethylases, catalyse the removal of m^6^A. Currently, emerging evidence has shown that transcription factor and histone modification signatures together shape m^6^A deposition, suggesting that the contribution of transcription factor histone modification contributes to the modulation of m^6^A. The interplay between histone H3K36me3 and m^6^A modification provides a novel layer of gene expression regulation. Further crosstalk between m^6^A RNA modification and other epigenetic modifications should be carefully elucidated in the future. Nutritional metabolism and metabolites also influence the regulation of m^6^A. It is possible that tissue-specific m^6^A levels partially correlate with the metabolic activities of specific organs. Overall, the precise regulatory mechanisms of m^6^A are still in their infancy and need to be further investigated. Although high-throughput sequencing-based techniques have greatly promoted the research field of m^6^A methylation, none of the available approaches have simultaneously reached the achievements with single-base resolution, quantification of m^6^A disposition, and low input of RNA. A future method for the direct sequencing of m^6^A RNA will be better for illustrating its functions and dynamics *in vivo*.

As mentioned above, abnormal m^6^A methylation is closely related to various diseases, including cancer, cardiovascular disease, metabolic syndrome, psychiatric disorders and autoinflammatory disorders. Accordingly, m^6^A methylation is a double-edged sword for tumour: a lack of m^6^A modification on specific genes accelerates tumour development, while over-m^6^A modification of other genes also induces tumour progression. In fact, many major challenges remain in elucidating the relationships between m^6^A and cancer. First, whether the multiple roles proposed in cancer actually rely on m^6^A modification should be considered. Second, we should not ignore the notion that m^6^A-related effectors may mediate tumour development and the progression in a m^6^A-independent manner. Third, it is worth noting that we elucidate the regulatory association of noncoding RNAs and m^6^A methylation in tumour. Fourth, the effects of m^6^A regulators on tumor cells and immune cells are complicated and need to be carefully concerned. Fifth, over 170 RNA modifications have been identified, it is worth evaluating whether other RNA modifications in the same RNA transcripts affects the role of m6A methylation in human diseases. Finally, further studies should be carried out to assess the clinical value of m^6^A in diseases.

Many studies have demonstrated that m^6^A regulatory factors are suitable as therapeutic targets, and some inhibitors of m^6^A-related factors, especially FTO, have been discovered. Nevertheless, no clinical trials using m^6^A inhibitors for the treatment of cancer and other diseases have been reported yet. We also provide several reasonable strategies for driving m^6^A-based therapy: 1) Nanoparticles can specifically deliver m^6^A modification molecules to target immune cells for tumour immunotherapeutic treatment. 2) A programmable m^6^A gene-editing system by dCas13 or dRCas9 provides a potential tool for the treatment of diseases. 3) Chimeric antigen receptor (CAR) immune cells with lentivirus-mediated gene delivery of m^6^A effectors are beneficial to cancer immunotherapy. 4) It is feasible to treat cancer and other diseases by using a m^6^A inhibitor combined with other therapies. Further studies are urgently required for the understanding of RNA m^6^A modifications and clinical applications.

## References

[B1] Aguilo F. Zhang F. Sancho A. Fidalgo M. Di Cecilia S. Vashisht A. (2015). Coordination of m(6)A mRNA methylation and gene transcription by ZFP217 regulates pluripotency and reprogramming. Cell Stem Cell 17 (6), 689–704. 10.1016/j.stem.2015.09.005 26526723 PMC4671830

[B2] Babic I. Anderson E. S. Tanaka K. Guo D. Masui K. Li B. (2013). EGFR mutation-induced alternative splicing of Max contributes to growth of glycolytic tumors in brain cancer. Cell Metab. 17 (6), 1000–1008. 10.1016/j.cmet.2013.04.013 23707073 PMC3679227

[B3] Bansal H. Yihua Q. Iyer S. P. Ganapathy S. Proia D. A. Penalva L. O. (2014). WTAP is a novel oncogenic protein in acute myeloid leukemia. Leukemia 28 (5), 1171–1174. 10.1038/leu.2014.16 24413322 PMC4369791

[B4] Barbieri I. Tzelepis K. Pandolfini L. Shi J. Millan-Zambrano G. Robson S. C. (2017). Promoter-bound METTL3 maintains myeloid leukaemia by m(6)A-dependent translation control. Nature 552 (7683), 126–131. 10.1038/nature24678 29186125 PMC6217924

[B5] Bawankar P. Lence T. Paolantoni C. Haussmann I. U. Kazlauskiene M. Jacob D. (2021). Hakai is required for stabilization of core components of the m(6)A mRNA methylation machinery. Nat. Commun. 12 (1), 3778. 10.1038/s41467-021-23892-5 34145251 PMC8213727

[B6] Bayoumi M. Rohaim M. A. Munir M. (2020). Structural and virus regulatory insights into avian N6-methyladenosine (m6A) machinery. Front. Cell Dev. Biol. 8, 543. 10.3389/fcell.2020.00543 32760718 PMC7373739

[B7] Bedi R. K. Huang D. Eberle S. A. Wiedmer L. Sledz P. Caflisch A. (2020). Small-molecule inhibitors of METTL3, the major human epitranscriptomic writer. ChemMedChem 15 (9), 744–748. 10.1002/cmdc.202000011 32159918

[B8] Bertero A. Brown S. Madrigal P. Osnato A. Ortmann D. Yiangou L. (2018). The SMAD2/3 interactome reveals that TGFβ controls m6A mRNA methylation in pluripotency. Nature 555 (7695), 256–259. 10.1038/nature25784 29489750 PMC5951268

[B9] Bi Z. Liu Y. Zhao Y. Yao Y. Wu R. Liu Q. (2019). A dynamic reversible RNA N(6) -methyladenosine modification: Current status and perspectives. J. Cell. Physiol. 234 (6), 7948–7956. 10.1002/jcp.28014 30644095

[B10] Bokar J. A. Rath-Shambaugh M. E. Ludwiczak R. Narayan P. Rottman F. (1994). Characterization and partial purification of mRNA N6-adenosine methyltransferase from HeLa cell nuclei. Internal mRNA methylation requires a multisubunit complex. J. Biol. Chem. 269 (26), 17697–17704. 10.1016/s0021-9258(17)32497-3 8021282

[B11] Cai J. Chen Z. Zhang Y. Wang J. Zhang Z. Wu J. (2022). CircRHBDD1 augments metabolic rewiring and restricts immunotherapy efficacy via m(6)A modification in hepatocellular carcinoma. Mol. Ther. Oncolytics 24, 755–771. 10.1016/j.omto.2022.02.021 35317519 PMC8908059

[B12] Chen K. Luo G. Z. He C. (2015). High-resolution mapping of N⁶-Methyladenosine in transcriptome and genome using a photo-crosslinking-assisted strategy. Methods Enzymol. 560, 161–185. 10.1016/bs.mie.2015.03.012 26253971

[B13] Cheng M. Sheng L. Gao Q. Xiong Q. Zhang H. Wu M. (2019). The m6A methyltransferase METTL3 promotes bladder cancer progression via AFF4/NF-κB/MYC signaling network. Oncogene 38 (19), 3667–3680. 10.1038/s41388-019-0683-z 30659266

[B14] Cui Q. Shi H. Ye P. Li L. Qu Q. Sun G. (2017). m(6)A RNA methylation regulates the self-renewal and tumorigenesis of glioblastoma stem cells. Cell Rep. 18 (11), 2622–2634. 10.1016/j.celrep.2017.02.059 28297667 PMC5479356

[B15] Dai F. Wu Y. Lu Y. An C. Zheng X. Dai L. (2020). Crosstalk between RNA m(6)A modification and non-coding RNA contributes to cancer growth and progression. Mol. Ther. Nucleic Acids 22, 62–71. 10.1016/j.omtn.2020.08.004 32911345 PMC7486578

[B16] De Jesus D. F. Zhang Z. Kahraman S. Brown N. K. Chen M. Hu J. (2019). m(6)A mRNA methylation regulates human beta-cell Biology in physiological states and in type 2 diabetes. Nat. Metab. 1 (8), 765–774. 10.1038/s42255-019-0089-9 31867565 PMC6924515

[B17] Deng J. Chen S. Wang F. Zhao H. Xie Z. Xu Z. (2016). Effects of hnRNP A2/B1 knockdown on inhibition of glioblastoma cell invasion, growth and survival. Mol. Neurobiol. 53 (2), 1132–1144. 10.1007/s12035-014-9080-3 25586062

[B18] Deng X. Chen K. Luo G. Z. Weng X. Ji Q. Zhou T. (2015). Widespread occurrence of N6-methyladenosine in bacterial mRNA. Nucleic Acids Res. 43 (13), 6557–6567. 10.1093/nar/gkv596 26068471 PMC4513869

[B19] Desrosiers R. Friderici K. Rottman F. (1974). Identification of methylated nucleosides in messenger RNA from Novikoff hepatoma cells. Proc. Natl. Acad. Sci. U. S. A. 71 (10), 3971–3975. 10.1073/pnas.71.10.3971 4372599 PMC434308

[B20] Ding L. Li J. Song B. Xiao X. Zhang B. Qi M. (2016). Curcumin rescues high fat diet-induced obesity and insulin sensitivity in mice through regulating SREBP pathway. Toxicol. Appl. Pharmacol. 304, 99–109. 10.1016/j.taap.2016.05.011 27208389

[B21] Dominissini D. Moshitch-Moshkovitz S. Schwartz S. Salmon-Divon M. Ungar L. Osenberg S. (2012). Topology of the human and mouse m6A RNA methylomes revealed by m6A-seq. Nature 485 (7397), 201–206. 10.1038/nature11112 22575960

[B22] Dong L. Chen C. Zhang Y. Guo P. Wang Z. Li J. (2021). The loss of RNA N(6)-adenosine methyltransferase Mettl14 in tumor-associated macrophages promotes CD8(+) T cell dysfunction and tumor growth. Cancer Cell 39 (7), 945–957. e910. 10.1016/j.ccell.2021.04.016 34019807

[B23] Dorn L. E. Lasman L. Chen J. Xu X. Hund T. J. Medvedovic M. (2019). The N(6)-methyladenosine mRNA methylase METTL3 controls cardiac homeostasis and hypertrophy. Circulation 139 (4), 533–545. 10.1161/CIRCULATIONAHA.118.036146 30586742 PMC6340720

[B24] Duncan T. M. Reed M. C. Nijhout H. F. (2013). The relationship between intracellular and plasma levels of folate and metabolites in the methionine cycle: A model. Mol. Nutr. Food Res. 57 (4), 628–636. 10.1002/mnfr.201200125 23143835 PMC3786706

[B25] Edens B. M. Vissers C. Su J. Arumugam S. Xu Z. Shi H. (2019). FMRP modulates neural differentiation through m(6)a-dependent mRNA nuclear export. Cell Rep. 28 (4), 845–854. 10.1016/j.celrep.2019.06.072 31340148 PMC6687293

[B26] Feng C. Liu Y. Wang G. Deng Z. Zhang Q. Wu W. (2014). Crystal structures of the human RNA demethylase Alkbh5 reveal basis for substrate recognition. J. Biol. Chem. 289 (17), 11571–11583. 10.1074/jbc.M113.546168 24616105 PMC4002068

[B27] Garcia-Campos M. A. Edelheit S. Toth U. Safra M. Shachar R. Viukov S. (2019). Deciphering the "m(6)A code" via antibody-independent quantitative profiling. Cell 178 (3), 731–747. e716. 10.1016/j.cell.2019.06.013 31257032

[B28] Gong P. J. Shao Y. C. Yang Y. Song W. J. He X. Zeng Y. F. (2020). Analysis of N6-methyladenosine methyltransferase reveals METTL14 and ZC3H13 as tumor suppressor genes in breast cancer. Front. Oncol. 10, 578963. 10.3389/fonc.2020.578963 33363011 PMC7757663

[B29] Gu C. Wang Z. Zhou N. Li G. Kou Y. Luo Y. (2019). Mettl14 inhibits bladder TIC self-renewal and bladder tumorigenesis through N(6)-methyladenosine of Notch1. Mol. Cancer 18 (1), 168. 10.1186/s12943-019-1084-1 31760940 PMC6876123

[B30] Guo J. Zheng J. Zhang H. Tong J. (2021). RNA m6A methylation regulators in ovarian cancer. Cancer Cell Int. 21 (1), 609. 10.1186/s12935-021-02318-8 34794452 PMC8600856

[B31] Han J. Wang J. Z. Yang X. Yu H. Zhou R. Lu H. C. (2019). METTL3 promote tumor proliferation of bladder cancer by accelerating pri-miR221/222 maturation in m6A-dependent manner. Mol. Cancer 18 (1), 110. 10.1186/s12943-019-1036-9 31228940 PMC6588935

[B32] Han M. Liu Z. Xu Y. Liu X. Wang D. Li F. (2020). Abnormality of m6A mRNA methylation is involved in alzheimer's disease. Front. Neurosci. 14, 98. 10.3389/fnins.2020.00098 32184705 PMC7058666

[B33] Haussmann I. U. Bodi Z. Sanchez-Moran E. Mongan N. P. Archer N. Fray R. G. (2016). m(6)A potentiates Sxl alternative pre-mRNA splicing for robust Drosophila sex determination. Nature 540 (7632), 301–304. 10.1038/nature20577 27919081

[B34] He P. C. He C. (2021). m(6) A RNA methylation: from mechanisms to therapeutic potential. EMBO J. 40 (3), e105977. 10.15252/embj.2020105977 33470439 PMC7849164

[B35] Hu B. B. Wang X. Y. Gu X. Y. Zou C. Gao Z. J. Zhang H. (2019). N(6)-methyladenosine (m(6)A) RNA modification in gastrointestinal tract cancers: Roles, mechanisms, and applications. Mol. Cancer 18 (1), 178. 10.1186/s12943-019-1099-7 31810483 PMC6898962

[B36] Hu L. Liu S. Peng Y. Ge R. Su R. Senevirathne C. (2022). m(6)A RNA modifications are measured at single-base resolution across the mammalian transcriptome. Nat. Biotechnol. 40, 1210–1219. 10.1038/s41587-022-01243-z 35288668 PMC9378555

[B37] Huang H. Weng H. Sun W. Qin X. Shi H. Wu H. (2018). Recognition of RNA N(6)-methyladenosine by IGF2BP proteins enhances mRNA stability and translation. Nat. Cell Biol. 20 (3), 285–295. 10.1038/s41556-018-0045-z 29476152 PMC5826585

[B38] Huang H. Weng H. Zhou K. Wu T. Zhao B. S. Sun M. (2019). Histone H3 trimethylation at lysine 36 guides m(6)A RNA modification co-transcriptionally. Nature 567 (7748), 414–419. 10.1038/s41586-019-1016-7 30867593 PMC6438714

[B39] Huang R. Zhang Y. Bai Y. Han B. Ju M. Chen B. (2020). N(6)-Methyladenosine modification of fatty acid amide hydrolase messenger RNA in circular RNA STAG1-regulated astrocyte dysfunction and depressive-like behaviors. Biol. Psychiatry 88 (5), 392–404. 10.1016/j.biopsych.2020.02.018 32387133

[B40] Huang Y. Yan J. Li Q. Li J. Gong S. Zhou H. (2015). Meclofenamic acid selectively inhibits FTO demethylation of m6A over ALKBH5. Nucleic Acids Res. 43 (1), 373–384. 10.1093/nar/gku1276 25452335 PMC4288171

[B41] Kang H. Zhang Z. Yu L. Li Y. Liang M. Zhou L. (2018). FTO reduces mitochondria and promotes hepatic fat accumulation through RNA demethylation. J. Cell. Biochem. 119 (7), 5676–5685. 10.1002/jcb.26746 29384213

[B42] Kasowitz S. D. Ma J. Anderson S. J. Leu N. A. Xu Y. Gregory B. D. (2018). Nuclear m6A reader YTHDC1 regulates alternative polyadenylation and splicing during mouse oocyte development. PLoS Genet. 14 (5), e1007412. 10.1371/journal.pgen.1007412 29799838 PMC5991768

[B43] Ke S. Alemu E. A. Mertens C. Gantman E. C. Fak J. J. Mele A. (2015). A majority of m6A residues are in the last exons, allowing the potential for 3' UTR regulation. Genes Dev. 29 (19), 2037–2053. 10.1101/gad.269415.115 26404942 PMC4604345

[B44] Keller L. Xu W. Wang H. X. Winblad B. Fratiglioni L. Graff C. (2011). The obesity related gene, FTO, interacts with APOE, and is associated with alzheimer's disease risk: A prospective cohort study. J. Alzheimers Dis. 23 (3), 461–469. 10.3233/JAD-2010-101068 21098976

[B45] Knuckles P. Lence T. Haussmann I. U. Jacob D. Kreim N. Carl S. H. (2018). Zc3h13/Flacc is required for adenosine methylation by bridging the mRNA-binding factor Rbm15/Spenito to the m(6)A machinery component Wtap/Fl(2)d. Genes Dev. 32 (5-6), 415–429. 10.1101/gad.309146.117 29535189 PMC5900714

[B46] Li F. Kennedy S. Hajian T. Gibson E. Seitova A. Xu C. (2016a). A radioactivity-based assay for screening human m6A-RNA methyltransferase, METTL3-METTL14 complex, and demethylase ALKBH5. J. Biomol. Screen. 21 (3), 290–297. 10.1177/1087057115623264 26701100

[B47] Li H. B. Tong J. Zhu S. Batista P. J. Duffy E. E. Zhao J. (2017a). m(6)A mRNA methylation controls T cell homeostasis by targeting the IL-7/STAT5/SOCS pathways. Nature 548 (7667), 338–342. 10.1038/nature23450 28792938 PMC5729908

[B48] Li N. Kang Y. Wang L. Huff S. Tang R. Hui H. (2020). ALKBH5 regulates anti-PD-1 therapy response by modulating lactate and suppressive immune cell accumulation in tumor microenvironment. Proc. Natl. Acad. Sci. U. S. A. 117 (33), 20159–20170. 10.1073/pnas.1918986117 32747553 PMC7443867

[B49] Li Q. Huang Y. Liu X. Gan J. Chen H. Yang C. G. (2016b). Rhein inhibits AlkB repair enzymes and sensitizes cells to methylated DNA damage. J. Biol. Chem. 291 (21), 11083–11093. 10.1074/jbc.M115.711895 27015802 PMC4900258

[B50] Li Z. Qian P. Shao W. Shi H. He X. C. Gogol M. (2018). Suppression of m(6)A reader Ythdf2 promotes hematopoietic stem cell expansion. Cell Res. 28 (9), 904–917. 10.1038/s41422-018-0072-0 30065315 PMC6123498

[B51] Li Z. Weng H. Su R. Weng X. Zuo Z. Li C. (2017b). FTO plays an oncogenic role in acute myeloid leukemia as a N(6)-methyladenosine RNA demethylase. Cancer Cell 31 (1), 127–141. 10.1016/j.ccell.2016.11.017 28017614 PMC5234852

[B52] Linder B. Grozhik A. V. Olarerin-George A. O. Meydan C. Mason C. E. Jaffrey S. R. (2015). Single-nucleotide-resolution mapping of m6A and m6Am throughout the transcriptome. Nat. Methods 12 (8), 767–772. 10.1038/nmeth.3453 26121403 PMC4487409

[B53] Liu J. Yue Y. Han D. Wang X. Fu Y. Zhang L. (2014). A METTL3-METTL14 complex mediates mammalian nuclear RNA N6-adenosine methylation. Nat. Chem. Biol. 10 (2), 93–95. 10.1038/nchembio.1432 24316715 PMC3911877

[B54] Liu L. Li H. Hu D. Wang Y. Shao W. Zhong J. (2022). Insights into N6-methyladenosine and programmed cell death in cancer. Mol. Cancer 21 (1), 32. 10.1186/s12943-022-01508-w 35090469 PMC8796496

[B55] Liu N. Pan T. (2016). Probing N⁶-methyladenosine (m⁶A) RNA modification in total RNA with SCARLET. Methods Mol. Biol. 1358, 285–292. 10.1007/978-1-4939-3067-8_17 26463390

[B56] Liu T. Wei Q. Jin J. Luo Q. Liu Y. Yang Y. (2020). The m6A reader YTHDF1 promotes ovarian cancer progression via augmenting EIF3C translation. Nucleic Acids Res. 48 (7), 3816–3831. 10.1093/nar/gkaa048 31996915 PMC7144925

[B57] Liu Y. Liang G. Xu H. Dong W. Dong Z. Qiu Z. (2021). Tumors exploit FTO-mediated regulation of glycolytic metabolism to evade immune surveillance. Cell Metab. 33 (6), 1221–1233. e1211. 10.1016/j.cmet.2021.04.001 33910046

[B58] Lo N. Xu X. Soares F. He H. H. (2022). The basis and promise of programmable RNA editing and modification. Front. Genet. 13, 834413. 10.3389/fgene.2022.834413 35154288 PMC8831800

[B59] Lu T. X. Zheng Z. Zhang L. Sun H. L. Bissonnette M. Huang H. (2020). A new model of spontaneous colitis in mice induced by deletion of an RNA m(6)A methyltransferase component METTL14 in T cells. Cell. Mol. Gastroenterol. Hepatol. 10 (4), 747–761. 10.1016/j.jcmgh.2020.07.001 32634481 PMC7498954

[B60] Luo Q. Fu B. Zhang L. Guo Y. Huang Z. Li J. (2020). Decreased peripheral blood ALKBH5 correlates with markers of autoimmune response in systemic lupus erythematosus. Dis. Markers 2020, 8193895. 10.1155/2020/8193895 32685056 PMC7334764

[B61] Ma H. Wang X. Cai J. Dai Q. Natchiar S. K. Lv R. (2019). N(6-)Methyladenosine methyltransferase ZCCHC4 mediates ribosomal RNA methylation. Nat. Chem. Biol. 15 (1), 88–94. 10.1038/s41589-018-0184-3 30531910 PMC6463480

[B62] Ma S. Yan J. Barr T. Zhang J. Chen Z. Wang L. S. (2021). The RNA m6A reader YTHDF2 controls NK cell antitumor and antiviral immunity. J. Exp. Med. 218 (8), e20210279. 10.1084/jem.20210279 34160549 PMC8225680

[B63] Ma X. X. Cao Z. G. Zhao S. L. (2020). m6A methyltransferase METTL3 promotes the progression of prostate cancer via m6A-modified LEF1. Eur. Rev. Med. Pharmacol. Sci. 24 (7), 3565–3571. 10.26355/eurrev_202004_20817 32329830

[B64] Malacrida A. Rivara M. Di Domizio A. Cislaghi G. Miloso M. Zuliani V. (2020). 3D proteome-wide scale screening and activity evaluation of a new ALKBH5 inhibitor in U87 glioblastoma cell line. Bioorg. Med. Chem. 28 (4), 115300. 10.1016/j.bmc.2019.115300 31937477

[B65] Mao Y. Dong L. Liu X. M. Guo J. Ma H. Shen B. (2019). m(6)A in mRNA coding regions promotes translation via the RNA helicase-containing YTHDC2. Nat. Commun. 10 (1), 5332. 10.1038/s41467-019-13317-9 31767846 PMC6877647

[B66] Mar B. G. Chu S. H. Kahn J. D. Krivtsov A. V. Koche R. Castellano C. A. (2017). SETD2 alterations impair DNA damage recognition and lead to resistance to chemotherapy in leukemia. Blood 130 (24), 2631–2641. 10.1182/blood-2017-03-775569 29018079 PMC5731084

[B67] Mauer J. Luo X. Blanjoie A. Jiao X. Grozhik A. V. Patil D. P. (2017). Reversible methylation of m(6)Am in the 5' cap controls mRNA stability. Nature 541 (7637), 371–375. 10.1038/nature21022 28002401 PMC5513158

[B68] Mauer J. Sindelar M. Despic V. Guez T. Hawley B. R. Vasseur J. J. (2019). FTO controls reversible m(6)Am RNA methylation during snRNA biogenesis. Nat. Chem. Biol. 15 (4), 340–347. 10.1038/s41589-019-0231-8 30778204 PMC6984009

[B69] Meyer K. D. Saletore Y. Zumbo P. Elemento O. Mason C. E. Jaffrey S. R. (2012). Comprehensive analysis of mRNA methylation reveals enrichment in 3' UTRs and near stop codons. Cell 149 (7), 1635–1646. 10.1016/j.cell.2012.05.003 22608085 PMC3383396

[B70] Moroz-Omori E. V. Huang D. Kumar Bedi R. Cheriyamkunnel S. J. Bochenkova E. Dolbois A. (2021). METTL3 inhibitors for epitranscriptomic modulation of cellular processes. ChemMedChem 16 (19), 3035–3043. 10.1002/cmdc.202100291 34237194 PMC8518639

[B71] Nagarajan A. Janostiak R. Wajapeyee N. (2019). Dot blot analysis for measuring global N(6)-methyladenosine modification of RNA. Methods Mol. Biol. 1870, 263–271. 10.1007/978-1-4939-8808-2_20 30539562

[B72] Nassar D. Blanpain C. (2016). Cancer stem cells: Basic concepts and therapeutic implications. Annu. Rev. Pathol. 11, 47–76. 10.1146/annurev-pathol-012615-044438 27193450

[B73] Nishizawa Y. Konno M. Asai A. Koseki J. Kawamoto K. Miyoshi N. (2018). Oncogene c-Myc promotes epitranscriptome m(6)A reader YTHDF1 expression in colorectal cancer. Oncotarget 9 (7), 7476–7486. 10.18632/oncotarget.23554 29484125 PMC5800917

[B74] Niu Y. Zhao X. Wu Y. S. Li M. M. Wang X. J. Yang Y. G. (2013). N6-methyl-adenosine (m6A) in RNA: An old modification with a novel epigenetic function. Genomics Proteomics Bioinforma. 11 (1), 8–17. 10.1016/j.gpb.2012.12.002 PMC4357660 23453015

[B75] Oerum S. Meynier V. Catala M. Tisne C. (2021). A comprehensive review of m6A/m6Am RNA methyltransferase structures. Nucleic Acids Res. 49 (13), 7239–7255. 10.1093/nar/gkab378 34023900 PMC8287941

[B76] Patil D. P. Chen C. K. Pickering B. F. Chow A. Jackson C. Guttman M. (2016). m(6)A RNA methylation promotes XIST-mediated transcriptional repression. Nature 537 (7620), 369–373. 10.1038/nature19342 27602518 PMC5509218

[B77] Pendleton K. E. Chen B. Liu K. Hunter O. V. Xie Y. Tu B. P. (2017). The U6 snRNA m(6)A methyltransferase METTL16 regulates SAM synthetase intron retention. Cell 169 (5), 824–835. e814. 10.1016/j.cell.2017.05.003 28525753 PMC5502809

[B78] Peng S. Xiao W. Ju D. Sun B. Hou N. Liu Q. (2019). Identification of entacapone as a chemical inhibitor of FTO mediating metabolic regulation through FOXO1. Sci. Transl. Med. 11 (488), eaau7116. 10.1126/scitranslmed.aau7116 30996080

[B79] Ping X. L. Sun B. F. Wang L. Xiao W. Yang X. Wang W. J. (2014). Mammalian WTAP is a regulatory subunit of the RNA N6-methyladenosine methyltransferase. Cell Res. 24 (2), 177–189. 10.1038/cr.2014.3 24407421 PMC3915904

[B80] Qiao Y. Yang Q. Song C. Chang J. (2017). Computational insights into the origin of decrease/increase in potency of N-CDPCB analogues toward FTO. J. Biomol. Struct. Dyn. 35 (8), 1758–1765. 10.1080/07391102.2016.1193445 27227432

[B81] Qiao Y. Zhou B. Zhang M. Liu W. Han Z. Song C. (2016). A novel inhibitor of the obesity-related protein FTO. Biochemistry 55 (10), 1516–1522. 10.1021/acs.biochem.6b00023 26915401

[B82] Saletore Y. Meyer K. Korlach J. Vilfan I. D. Jaffrey S. Mason C. E. (2012). The birth of the epitranscriptome: Deciphering the function of RNA modifications. Genome Biol. 13 (10), 175. 10.1186/gb-2012-13-10-175 23113984 PMC3491402

[B83] Scholler E. Weichmann F. Treiber T. Ringle S. Treiber N. Flatley A. (2018). Interactions, localization, and phosphorylation of the m(6)A generating METTL3-METTL14-WTAP complex. RNA 24 (4), 499–512. 10.1261/rna.064063.117 29348140 PMC5855951

[B84] Selberg S. Blokhina D. Aatonen M. Koivisto P. Siltanen A. Mervaala E. (2019). Discovery of small molecules that activate RNA methylation through cooperative binding to the METTL3-14-WTAP complex active site. Cell Rep. 26 (13), 3762–3771. e3765. 10.1016/j.celrep.2019.02.100 30917327

[B85] Shi H. Wei J. He C. (2019). Where, when, and how: Context-dependent functions of RNA methylation writers, readers, and erasers. Mol. Cell 74 (4), 640–650. 10.1016/j.molcel.2019.04.025 31100245 PMC6527355

[B86] Shima H. Matsumoto M. Ishigami Y. Ebina M. Muto A. Sato Y. (2017). S-adenosylmethionine synthesis is regulated by selective N(6)-adenosine methylation and mRNA degradation involving METTL16 and YTHDC1. Cell Rep. 21 (12), 3354–3363. 10.1016/j.celrep.2017.11.092 29262316

[B87] Shu X. Cao J. Cheng M. Xiang S. Gao M. Li T. (2020). A metabolic labeling method detects m(6)A transcriptome-wide at single base resolution. Nat. Chem. Biol. 16 (8), 887–895. 10.1038/s41589-020-0526-9 32341503

[B88] Song H. Song J. Cheng M. Zheng M. Wang T. Tian S. (2021). METTL3-mediated m(6)A RNA methylation promotes the anti-tumour immunity of natural killer cells. Nat. Commun. 12 (1), 5522. 10.1038/s41467-021-25803-0 34535671 PMC8448775

[B89] Su R. Dong L. Li C. Nachtergaele S. Wunderlich M. Qing Y. (2018). R-2HG exhibits anti-tumor activity by targeting FTO/m(6)A/MYC/CEBPA signaling. Cell 172 (1-2), 90–105. 10.1016/j.cell.2017.11.031 29249359 PMC5766423

[B90] Taketo K. Konno M. Asai A. Koseki J. Toratani M. Satoh T. (2018). The epitranscriptome m6A writer METTL3 promotes chemo- and radioresistance in pancreatic cancer cells. Int. J. Oncol. 52 (2), 621–629. 10.3892/ijo.2017.4219 29345285

[B91] Thuring K. Schmid K. Keller P. Helm M. (2017). LC-MS analysis of methylated RNA. Methods Mol. Biol. 1562, 3–18. 10.1007/978-1-4939-6807-7_1 28349450

[B92] Tung Y. C. Ayuso E. Shan X. Bosch F. O'Rahilly S. Coll A. P. (2010). Hypothalamic-specific manipulation of Fto, the ortholog of the human obesity gene FTO, affects food intake in rats. PLoS One 5 (1), e8771. 10.1371/journal.pone.0008771 20098739 PMC2808248

[B93] van Tran N. Ernst F. G. M. Hawley B. R. Zorbas C. Ulryck N. Hackert P. (2019). The human 18S rRNA m6A methyltransferase METTL5 is stabilized by TRMT112. Nucleic Acids Res. 47 (15), 7719–7733. 10.1093/nar/gkz619 31328227 PMC6735865

[B94] Visvanathan A. Patil V. Arora A. Hegde A. S. Arivazhagan A. Santosh V. (2018). Essential role of METTL3-mediated m(6)A modification in glioma stem-like cells maintenance and radioresistance. Oncogene 37 (4), 522–533. 10.1038/onc.2017.351 28991227

[B95] Vu L. P. Pickering B. F. Cheng Y. Zaccara S. Nguyen D. Minuesa G. (2017). The N(6)-methyladenosine (m(6)A)-forming enzyme METTL3 controls myeloid differentiation of normal hematopoietic and leukemia cells. Nat. Med. 23 (11), 1369–1376. 10.1038/nm.4416 28920958 PMC5677536

[B96] Walcher L. Kistenmacher A. K. Suo H. Kitte R. Dluczek S. Strauss A. (2020). Cancer stem cells-origins and biomarkers: Perspectives for targeted personalized therapies. Front. Immunol. 11, 1280. 10.3389/fimmu.2020.01280 32849491 PMC7426526

[B97] Wang H. Zuo H. Liu J. Wen F. Gao Y. Zhu X. (2018a). Loss of YTHDF2-mediated m(6)A-dependent mRNA clearance facilitates hematopoietic stem cell regeneration. Cell Res. 28 (10), 1035–1038. 10.1038/s41422-018-0082-y 30150673 PMC6170435

[B98] Wang L. Song C. Wang N. Li S. Liu Q. Sun Z. (2020a). NADP modulates RNA m(6)A methylation and adipogenesis via enhancing FTO activity. Nat. Chem. Biol. 16 (12), 1394–1402. 10.1038/s41589-020-0601-2 32719557

[B99] Wang P. Doxtader K. A. Nam Y. (2016). Structural basis for cooperative function of Mettl3 and Mettl14 methyltransferases. Mol. Cell 63 (2), 306–317. 10.1016/j.molcel.2016.05.041 27373337 PMC4958592

[B100] Wang R. Han Z. Liu B. Zhou B. Wang N. Jiang Q. (2018b). Identification of natural compound radicicol as a potent FTO inhibitor. Mol. Pharm. 15 (9), 4092–4098. 10.1021/acs.molpharmaceut.8b00522 30063141

[B101] Wang T. Hong T. Huang Y. Su H. Wu F. Chen Y. (2015). Fluorescein derivatives as bifunctional molecules for the simultaneous inhibiting and labeling of FTO protein. J. Am. Chem. Soc. 137 (43), 13736–13739. 10.1021/jacs.5b06690 26457839

[B102] Wang Y. Xiao Y. Dong S. Yu Q. Jia G. (2020b). Antibody-free enzyme-assisted chemical approach for detection of N(6)-methyladenosine. Nat. Chem. Biol. 16 (8), 896–903. 10.1038/s41589-020-0525-x 32341502

[B103] Wang Y. Zeng L. Liang C. Zan R. Ji W. Zhang Z. (2019). Integrated analysis of transcriptome-wide m(6)A methylome of osteosarcoma stem cells enriched by chemotherapy. Epigenomics 11 (15), 1693–1715. 10.2217/epi-2019-0262 31650864

[B104] Wei C. M. Gershowitz A. Moss B. (1975). Methylated nucleotides block 5' terminus of HeLa cell messenger RNA. Cell 4 (4), 379–386. 10.1016/0092-8674(75)90158-0 164293

[B105] Wei J. Liu F. Lu Z. Fei Q. Ai Y. He P. C. (2018). Differential m(6)A, m(6)Am, and m(1)A demethylation mediated by FTO in the cell nucleus and cytoplasm. Mol. Cell 71 (6), 973–985. e975. 10.1016/j.molcel.2018.08.011 30197295 PMC6151148

[B106] Wen J. Lv R. Ma H. Shen H. He C. Wang J. (2018). Zc3h13 regulates nuclear RNA m(6)A methylation and mouse embryonic stem cell self-renewal. Mol. Cell 69 (6), 1028–1038. e1026. 10.1016/j.molcel.2018.02.015 29547716 PMC5858226

[B107] Weng H. Huang H. Wu H. Qin X. Zhao B. S. Dong L. (2018). METTL14 inhibits hematopoietic stem/progenitor differentiation and promotes leukemogenesis via mRNA m(6)A modification. Cell Stem Cell 22 (2), 191–205. e199. 10.1016/j.stem.2017.11.016 29290617 PMC5860916

[B108] Wiener D. Schwartz S. (2021). The epitranscriptome beyond m(6)A. Nat. Rev. Genet. 22 (2), 119–131. 10.1038/s41576-020-00295-8 33188361

[B109] Wilson C. Chen P. J. Miao Z. Liu D. R. (2020). Programmable m(6)A modification of cellular RNAs with a Cas13-directed methyltransferase. Nat. Biotechnol. 38 (12), 1431–1440. 10.1038/s41587-020-0572-6 32601430 PMC7718427

[B110] Wu J. Li Y. Yu J. Gan Z. Wei W. Wang C. (2020). Resveratrol attenuates high-fat diet induced hepatic lipid homeostasis disorder and decreases m(6)A RNA methylation. Front. Pharmacol. 11, 568006. 10.3389/fphar.2020.568006 33519432 PMC7845411

[B111] Wu R. Li A. Sun B. Sun J. G. Zhang J. Zhang T. (2019). A novel m(6)A reader Prrc2a controls oligodendroglial specification and myelination. Cell Res. 29 (1), 23–41. 10.1038/s41422-018-0113-8 30514900 PMC6318280

[B112] Xiao W. Adhikari S. Dahal U. Chen Y. S. Hao Y. J. Sun B. F. (2016). Nuclear m(6)A reader YTHDC1 regulates mRNA splicing. Mol. Cell 61 (4), 507–519. 10.1016/j.molcel.2016.01.012 26876937

[B113] Xie H. Li J. Ying Y. Yan H. Jin K. Ma X. (2020). METTL3/YTHDF2 m(6) A axis promotes tumorigenesis by degrading SETD7 and KLF4 mRNAs in bladder cancer. J. Cell. Mol. Med. 24 (7), 4092–4104. 10.1111/jcmm.15063 32126149 PMC7171394

[B114] Yadav P. K. Rajasekharan R. (2018). The m(6)A methyltransferase Ime4 and mitochondrial functions in yeast. Curr. Genet. 64 (2), 353–357. 10.1007/s00294-017-0758-8 28975387

[B115] Yang F. Jin H. Que B. Chao Y. Zhang H. Ying X. (2019). Dynamic m(6)A mRNA methylation reveals the role of METTL3-m(6)A-CDCP1 signaling axis in chemical carcinogenesis. Oncogene 38 (24), 4755–4772. 10.1038/s41388-019-0755-0 30796352 PMC6756049

[B116] Yang X. Wei X. Yang J. Du T. Yin C. Fu B. (2021). An unexpected role for Dicer as a reader of the unacetylated DNA binding domain of p53 in transcriptional regulation. Sci. Adv. 7 (19), eabi6684. 10.1126/sciadv.abi6684 34705508 PMC8550248

[B117] Yang Y. Wei Q. Tang Y. Yuanyuan W. Luo Q. Zhao H. (2020). Loss of hnRNPA2B1 inhibits malignant capability and promotes apoptosis via down-regulating Lin28B expression in ovarian cancer. Cancer Lett. 475, 43–52. 10.1016/j.canlet.2020.01.029 32006618

[B118] Yao Y. Yang Y. Guo W. Xu L. You M. Zhang Y. C. (2021). METTL3-dependent m(6)A modification programs T follicular helper cell differentiation. Nat. Commun. 12 (1), 1333. 10.1038/s41467-021-21594-6 33637761 PMC7910450

[B119] Yin H. Zhang X. Yang P. Zhang X. Peng Y. Li D. (2021). RNA m6A methylation orchestrates cancer growth and metastasis via macrophage reprogramming. Nat. Commun. 12 (1), 1394. 10.1038/s41467-021-21514-8 33654093 PMC7925544

[B120] Yu F. Wei J. Cui X. Yu C. Ni W. Bungert J. (2021a). Post-translational modification of RNA m6A demethylase ALKBH5 regulates ROS-induced DNA damage response. Nucleic Acids Res. 49 (10), 5779–5797. 10.1093/nar/gkab415 34048572 PMC8191756

[B121] Yu Q. Liu S. Yu L. Xiao Y. Zhang S. Wang X. (2021b). RNA demethylation increases the yield and biomass of rice and potato plants in field trials. Nat. Biotechnol. 39 (12), 1581–1588. 10.1038/s41587-021-00982-9 34294912

[B122] Yue H. Nie X. Yan Z. Weining S. (2019). N6-methyladenosine regulatory machinery in plants: Composition, function and evolution. Plant Biotechnol. J. 17 (7), 1194–1208. 10.1111/pbi.13149 31070865 PMC6576107

[B123] Yue Y. Liu J. Cui X. Cao J. Luo G. Zhang Z. (2018). VIRMA mediates preferential m(6)A mRNA methylation in 3'UTR and near stop codon and associates with alternative polyadenylation. Cell Discov. 4, 10. 10.1038/s41421-018-0019-0 29507755 PMC5826926

[B124] Zhang Q. Xu K. (Forthcoming 2022). The role of regulators of RNA m6A methylation in lung cancer. Genes & Dis. 10.1016/j.gendis.2021.12.017 PMC10201596 37223516

[B125] Zhang S. Zhao B. S. Zhou A. Lin K. Zheng S. Lu Z. (2017). m(6)A demethylase ALKBH5 maintains tumorigenicity of glioblastoma stem-like cells by sustaining FOXM1 expression and cell proliferation program. Cancer Cell 31 (4), 591–606. e596. 10.1016/j.ccell.2017.02.013 28344040 PMC5427719

[B126] Zhang X. Wei L. H. Wang Y. Xiao Y. Liu J. Zhang W. (2019a). Structural insights into FTO's catalytic mechanism for the demethylation of multiple RNA substrates. Proc. Natl. Acad. Sci. U. S. A. 116 (8), 2919–2924. 10.1073/pnas.1820574116 30718435 PMC6386707

[B127] Zhang Y. Kang M. Zhang B. Meng F. Song J. Kaneko H. (2019b). m(6)A modification-mediated CBX8 induction regulates stemness and chemosensitivity of colon cancer via upregulation of LGR5. Mol. Cancer 18 (1), 185. 10.1186/s12943-019-1116-x 31849331 PMC6918584

[B128] Zhang Z. Chen L. Q. Zhao Y. L. Yang C. G. Roundtree I. A. Zhang Z. (2019c). Single-base mapping of m(6)A by an antibody-independent method. Sci. Adv. 5 (7), eaax0250. 10.1126/sciadv.aax0250 31281898 PMC6609220

[B129] Zhao B. S. Wang X. Beadell A. V. Lu Z. Shi H. Kuuspalu A. (2017). m(6)A-dependent maternal mRNA clearance facilitates zebrafish maternal-to-zygotic transition. Nature 542 (7642), 475–478. 10.1038/nature21355 28192787 PMC5323276

[B130] Zhao Y. Shi Y. Shen H. Xie W. (2020). m(6)A-binding proteins: the emerging crucial performers in epigenetics. J. Hematol. Oncol. 13 (1), 35. 10.1186/s13045-020-00872-8 32276589 PMC7146974

[B131] Zhou B. Liu C. Xu L. Yuan Y. Zhao J. Zhao W. (2021a). N(6) -methyladenosine reader protein YT521-B homology domain-containing 2 suppresses liver steatosis by regulation of mRNA stability of lipogenic genes. Hepatology 73 (1), 91–103. 10.1002/hep.31220 32150756

[B132] Zhou H. Yin K. Zhang Y. Tian J. Wang S. (2021b). The RNA m6A writer METTL14 in cancers: Roles, structures, and applications. Biochim. Biophys. Acta. Rev. Cancer 1876 (2), 188609. 10.1016/j.bbcan.2021.188609 34375716

[B133] Zhou X. Chen J. Chen J. Wu W. Wang X. Wang Y. (2015). The beneficial effects of betaine on dysfunctional adipose tissue and N6-methyladenosine mRNA methylation requires the AMP-activated protein kinase α1 subunit. J. Nutr. Biochem. 26 (12), 1678–1684. 10.1016/j.jnutbio.2015.08.014 26365580

[B134] Zhou X. L. Huang F. J. Li Y. Huang H. Wu Q. C. (2021c). SEDT2/METTL14-mediated m6A methylation awakening contributes to hypoxia-induced pulmonary arterial hypertension in mice. Aging (Albany NY) 13 (5), 7538–7548. 10.18632/aging.202616 33658391 PMC7993666

[B135] Zhu W. Wang J. Z. Wei J. F. Lu C. (2021). Role of m6A methyltransferase component VIRMA in multiple human cancers (Review). Cancer Cell Int. 21 (1), 172. 10.1186/s12935-021-01868-1 33731118 PMC7968318

